# Synchrotron Ultraviolet Microspectroscopy on Rat Cortical Bone: Involvement of Tyrosine and Tryptophan in the Osteocyte and Its Environment

**DOI:** 10.1371/journal.pone.0043930

**Published:** 2012-08-28

**Authors:** Stéphane Pallu, Gael Y. Rochefort, Christelle Jaffre, Matthieu Refregiers, Delphine B. Maurel, Delphine Benaitreau, Eric Lespessailles, Frédéric Jamme, Christine Chappard, Claude-Laurent Benhamou

**Affiliations:** 1 Institut National de la Santé et de la Recherche Médicale, INSERM U-658, Université d’Orléans, Orléans, France; 2 DISCO beamline, Synchrotron SOLEIL, Gif-sur-Yvette, France; 3 Institut National de la Recherche Agronomique, INRA, unité U1008 Caractérisation et Elaboration des Produits Issus de l’Agriculture, CEPIA, Nantes, France; 4 Unité Mixte de Recherche Biomécanique et Biomatériaux Ostéo-Articulaires, B2OA UMR 7052, Centre National de la Recherche Scientifique, CNRS, Paris, France; Clermont Université, France

## Abstract

Alcohol induced osteoporosis is characterized by a bone mass decrease and microarchitecture alterations. Having observed an excess in osteocyte apoptosis, we aimed to assess the bone tissue biochemistry, particularly in the osteocyte and its environment. For this purpose, we used a model of alcohol induced osteoporosis in rats. Bone sections of cortical bone were investigated using synchrotron UV-microspectrofluorescence at subcellular resolution. We show that bone present three fluorescence peaks at 305, 333 and 385 nm, respectively corresponding to tyrosine, tryptophan and collagen. We have determined that tyrosine/collagen and tryptophan/collagen ratios were higher in the strong alcohol consumption group. Tryptophan is related to the serotonin metabolism involved in bone formation, while tyrosine is involved in the activity of tyrosine kinases and phosphatases in osteocytes. Our experiment represents the first combined synchrotron UV microspectroscopy analysis of bone tissue with a quantitative biochemical characterization in the osteocyte and surrounding matrix performed separately.

## Introduction

Spectroscopy-based approaches are essential in addressing the chemical composition and distribution of components across a biological tissue sample. This opens the opportunity to investigate the chemical changes occuring in the course of disease-associated lesions of biological tissue [1], [2]. For the recent past years, infrared microspectroscopy has been widely used in various biological studies [3]. Deep Ultraviolet (DUV) microspectroscopy is used for investigating autofluorescent components of cells and tissues [4]. Indeed, several metabolic markers do provide characteristic fluorescence emission spectra when excited with UV (NADH, collagen, tyrosine, tryptophan, lipo-pigments, elastin, pyridoxins) [5], [6]. Their localization can be determined, and the changes in molecular environment or oxidation are often associated with changes of the local fluorescence emission spectra [6]. As collagen constitutes the main protein of the organic matrix compartment of bone tissue, we have chosen to represent tyrosine and tryptophan amounts by ratios related to collagen level because the autofluorescence spectra are close and their maximal intensity may be impacted by overlapping incoming from the others spectra. The evaluation of the relative amount of both amino-acids was also performed. Because a continuous DUV source of photons with high spectral resolution is needed at microscopic scale, we used a synchrotron UV microspectrofluorimeter [7] setup to study our samples.

The main advantage of synchrotron UV imaging is that it does not necessitate any matrix deposition, embedding or staining to acquire images. Furthermore, the spatial resolution of this imaging technique yields information at the cellular level and permits to distinguish between cells and their surrounding matrix. Moreover, the main advantage of the synchrotron radiation is the high brightness of the source of photons, perfectly focalised onto the objective entrance lens. The interest of the association between synchrotron radiation and deep ultraviolet microspectroscopy has been previously demonstrated in liver and endocarditis vegetation [8], [9].

The interest of Synchrotron UV microspectroscopy to characterize the osteocytes and surrounding matrix has been investigated in this study and use to demonstrate the capabilities of UV spectroscopy method, particularly for biomedical research applications. Indeed, biochemical characterization of the osteocytes and their matrix present a real interest since these cells are considered as the orchestrator of the bone remodeling [10], [11]. We used a model of alcoholic induced osteoporosis which is linked to a decrease in bone formation [12] and an increase in bone resorption [13]. Some publications have previously shown systemic modulation of tyrosine and tryptophan in alcoholism [14], [15].

The amino-acids tryptophan and tyrosine are probably involved in the physiopathological process of this osteoporosis model. Indeed, recent works highlight the role of tryptophan in different bone metabolic pathways [16]–[19]. Measurement of the tryptophan/collagen ratio *in situ* could give informations related to serotonin, vitamin D receptor [17], [18] and PTHrP metabolisms [20], [21]. The role of the residue tyrosine in osteoporosis models has not been well documented to date. However, Protein Tyrosine Kinases/Protein Tyrosine Phosphatases are involved in the regulation of bone formation. Moreover, recent publications have shown that the protein tyrosine kinases activation is associated with alcohol abuse or dependence [22]–[24].

In the present study, two groups of male Wistar rats demonstrating alcohol-induced osteoporosis cases (separated by strong and moderate chronic consumption) were compared with control bones to study autofluorescence components such as tryptophan, tyrosine and collagen under synchrotron UV excitation. Having previously demonstrated a large excess of osteocyte apoptosis in this experiment [25], the interest of Synchrotron UV microspectroscopy to characterize the osteocyte and surrounding matrix biochemical composition separately has been investigated in this study.

## Methods

### Animals

The subjects were 27 male Wistar rats (Elevage Janvier, Le Genet-St-Isle, France) acclimatized for 2 weeks and maintained under constant temperature (21±2°C) and under 12 h/12 h light-dark cycles during the experiment. The rats were housed in groups of two per standard cage and provided with a commercial standard diet (M20, SDS, Dietex, St Gratien, France).

### Alcohol Treatment

The rats were 8 weeks old at baseline. At eleven weeks of age, the rats were chosen at random and assigned to one of the three following groups: Controls (C) (n = 11), ethanol at 35% v/v (A35) (n = 8) or ethanol at 25% v/v (A25) (n = 8). The protocol lasted 19 weeks, including 2 weeks of acclimation at the beginning. Then, the rats of the A25 and A35 group drank *ad libitum* a solution composed of ethanol and water for 17 weeks. The percentage of ethanol in the solution was progressively increased from 8% v/v to 35% by increasing every 3 days by 3% the amount of ethanol. This has been realized over 3 weeks. The food and the beverage were separated in order to better mimic the human drinking pattern. The quantity of food eaten was controlled in order to obtain the same daily calorie intake in the three groups. The procedure for the care and sacrifice of the animals was in accordance with the European Community standards on the care and use of laboratory animals (Ministère de l’agriculture, France, Authorisation INSERM45-001).

At the end of the study, all rats were anesthetised with pentobarbital sodium (0.1 ml per 100 g of body weight) and then killed by cardiac exsanguinations. After the death, tibias were dissected free of connective and fat tissue. They were fixed in a 4% v/v formalin solution and kept at +4°C.

### Bone Explants Preparation

As required the tibias were cut transversally in slices (thickness 200 µm) in the superior third part of the diaphysis, with a high speed rotary tool (Dremel 300, Dremel, USA).

### Bone Mineral Density Measurements

*In vivo* bone mineral density (BMD) of the left femur and whole body were measured in the A35, A25 and C groups at 10 and 19 weeks of age by dual-energy X-ray absorptiometry (DXA) using a Discovery Hologic apparatus adapted to small animals. The root-mean square coefficient of variation of *in vivo* whole body BMD was 0.87% and was determined from two repeated measures with repositioning on 27 animals.

### Morphological and Topological Characteristics of the Trabecular Bone

The microarchitecture of the femoral bone metaphysis was studied post-mortem using computed microtomography (µCT) (Skyscan 1072, Skyscan, Aartselaar, Belgium) for the A35, A25 and C groups. We chose this subregion because it is rich in trabecular bone. The characteristics and methods have already been described elsewhere [26]. The X-ray source was set at 70 kV and 100 µA, with an isotropic pixel size of 11 µm.

Two hundred and twenty-five slices were selected in the metaphysis under the cartilaginous bone. Simple global thresholding methods were used. The trabecular bone region of interest (ROI) was drawn with the free hand tool with “CT analyzer” software (Skyscan). The bone volume fraction BV/TV (%) and the trabecular thickness Tb.Th (mm) were extracted from the reconstructed tomogram [27].

### Morphological Characteristics of Cortical Bone

Cortical bone was characterized in the femoral metaphysis using post mortem µCT. The characteristics and methods have already been described previously [28], [29]. We analyzed the cortical porosity Ct. Po (%) (BV/TV equivalent), the pore number Po.N (1/mm) (Tb.N equivalent), the pore spacing Po.Sp (mm) (Tb.Sp equivalent) and the pore surface on the cortical volume PoS/CtV (mm^2^/mm^3^) (BS/TV equivalent). Given the imaging pixel size of 11 µm, the pores are generally considered to be Haversian and Volkmann canals, as described by Zebaze [30]. Cortical thickness Ct.Th (mm) was obtained by measuring the internal and external diameter on the medio-lateral axis of the femur with a caliper rule (Mitutoyo, Japan) and then by substracting the internal to the external diameter.

### Synchrotron-UV Microspectroscopy

**Figure 1:**
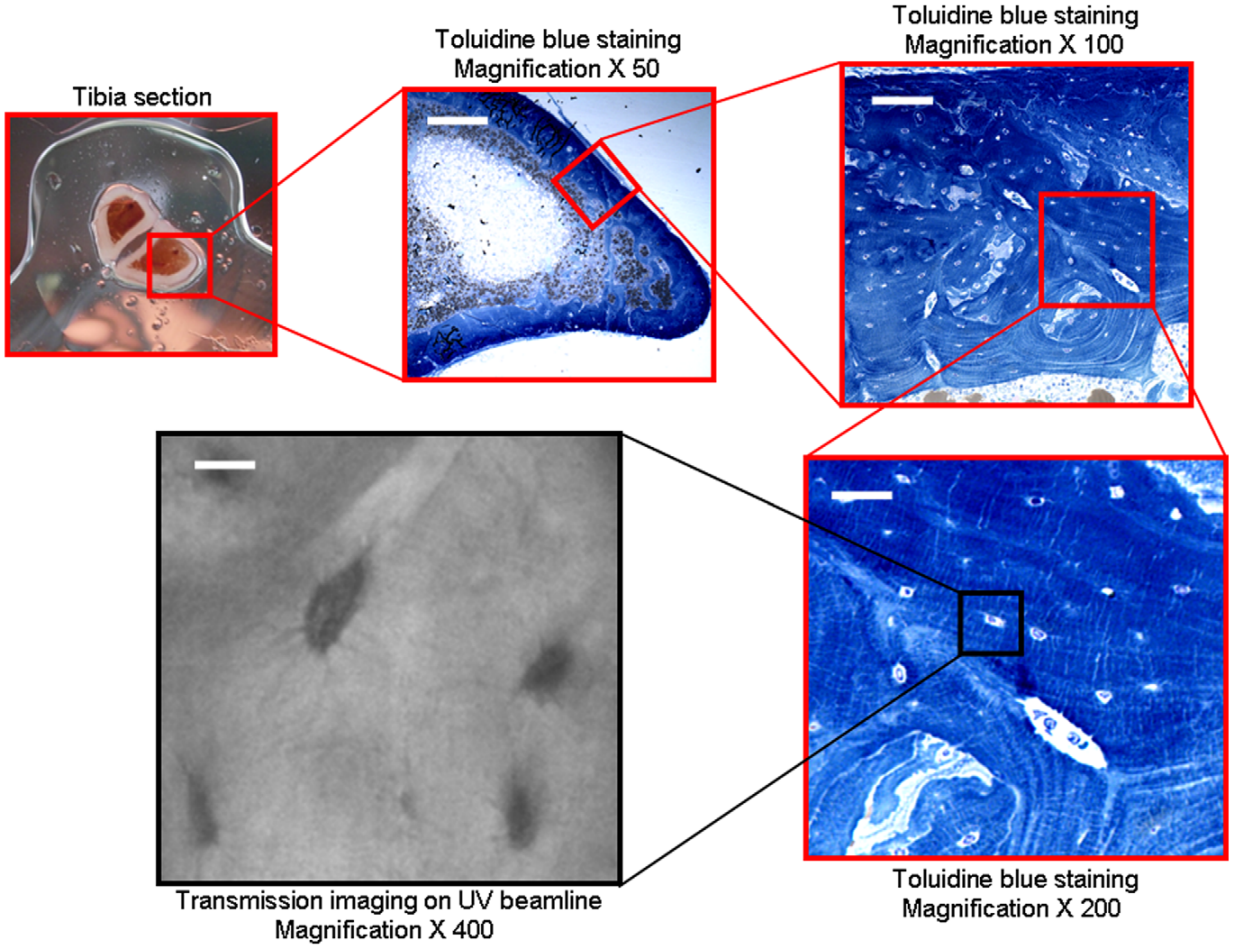
Bone sample ROI investigated on transmission image obtained on Synchrotron UV beamline. Tibia sections of 300 µm were cut. Magnifications (×50, ×100, ×200) represent histologic section after toluidine blue staining (white scale bars 80, 40, 20 µm, respectively). Magnification ×400 represents visible confocal microscopy acquired on UV beamline (the white scale bar indicates 10 µm).

**Figure 2:**
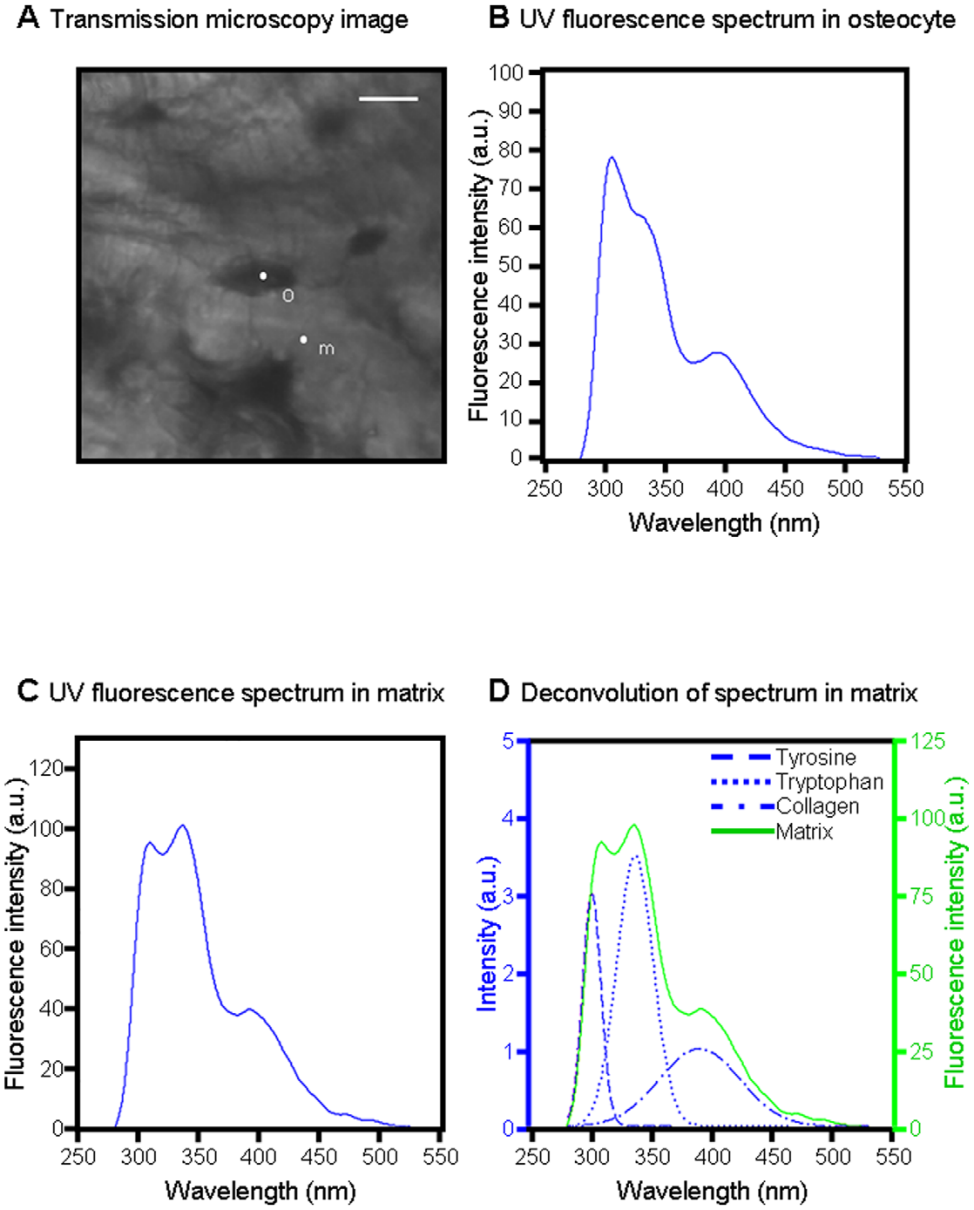
UV spectroscopy results from osteocyte and surrounding matrix. **A**: Transmission microscopy image showing ROIs, white scale bar 10 µm. **B**: UV fluorescence spectrum originating from osteocyte ROI pixel “o”. **C**: UV fluorescence spectrum originating from matrix ROI pixel “m”. **D**: Deconvolution of spectrum “m” into three Gaussians corresponding to tyrosine (a1), tryptophan (a2), and collagen (a3).

Synchrotron UV microspectroscopy was performed at the DISCO beamline at the SOLEIL synchrotron radiation facility (Saint-Aubin, France) [31]. Monochromatized UV light (typically between 270 and 330 nm) was used to excite tissue sections through a 40× ultrafluar objective (Ultrafluar, Zeiss, Germany). An example of the Regions of Interest (ROIs) investigated with UV beamline is indicated on figure **1**. The fluorescence emission spectrum arising from each excited pixel is recorded. Raster-scanning of the sample allows one to record x, y, λ, I maps of interest [7]. Mapping of 20×20 µm^2^ was performed with a 2×2 µm step size: the step size was chosen to provide a field of view containing several cells with a 5 s acquisition time per spectrum. Regions of Interest (ROIs) were selected, both centered around an osteocyte, and in the surrounding matrix (figure **1**). For each rat sample, 3 maps were measured, containing both osteocyte ROIs and matrix ROIs. Each map was therefore a matrix of 100 spectra from which noise and spikes were removed using a home-made routine in MATLAB (Mathworks Inc) available at DISCO beamline. The spectra were classified into subsets corresponding to osteocyte or matrix, using masks created from the transmission images. Thereafter, all spectra from all ROIs were loaded in Igor Pro (Wavemetrics Inc), the baselines were zeroed and the integral of the three peaks were measured as follows: area 1 for tyrosine, 290 to 317 nm (figure **2**), area 2 for tryptophan, 322 to 370 nm (figure **2**), area 3 for collagen, 380 to 440 nm (figure **2**). The collagen signal is originating from the crosslinks between the amino-acids.

**Figure 3:**
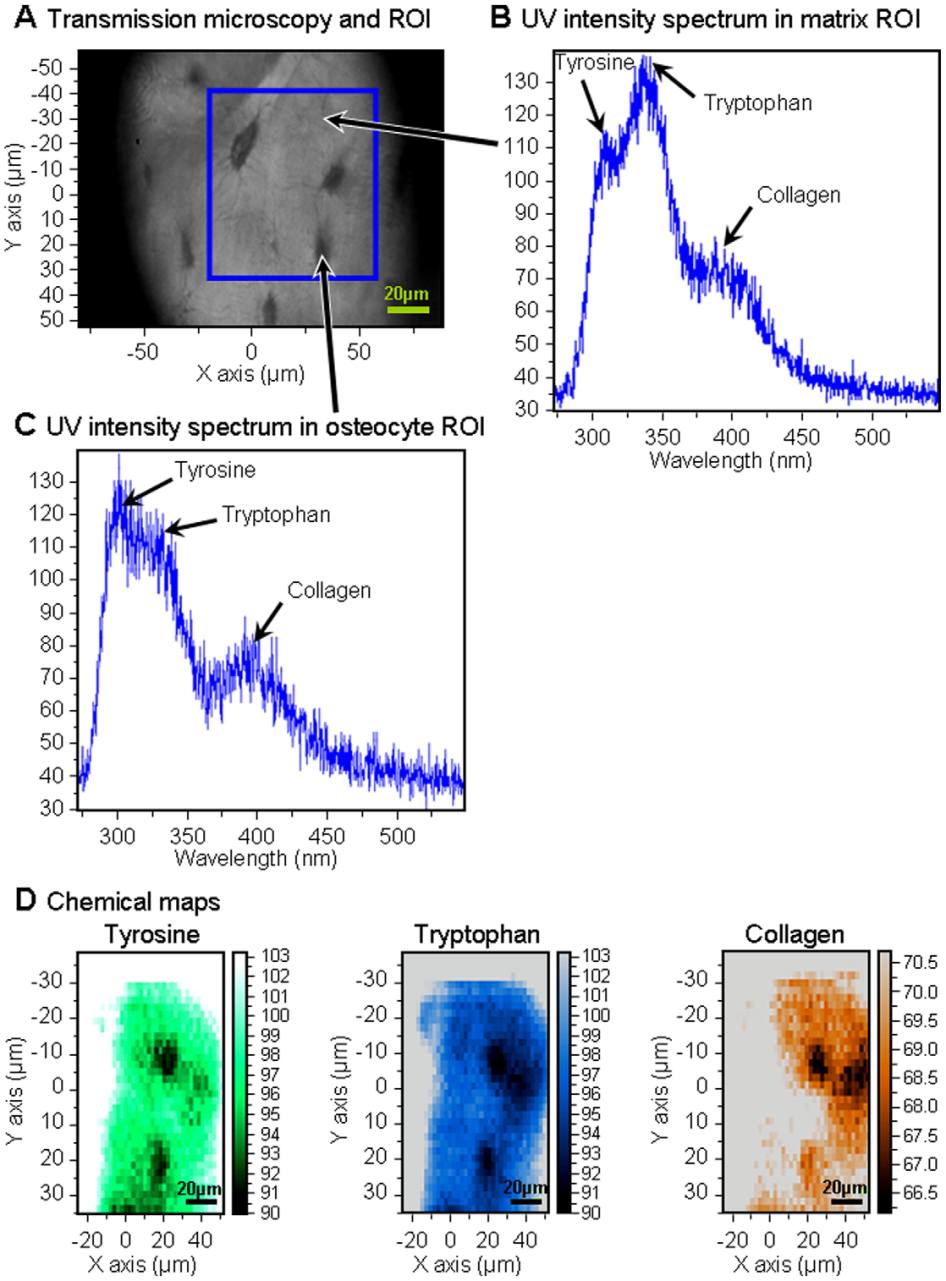
UV spectroscopy results from osteocyte and surrounding matrix ROI. **A**: Transmission microscopy image showing ROIs. **B**: UV intensity spectrum originating from one matrix ROI pixel (indicated with an arrow). **C**: UV intensity spectrum originating from one osteocyte ROI pixel as shown. **D**: UV chemical maps of osteocytes and surrounding matrix showing the tyrosine (green), tryptophan (blue), collagen (orange) distribution within the blue square marked in (a).

For each mapping, the ratios Tyrosine/Collagen (Tyr/Coll), Tryptophan/Collagen (Trp/Coll) and Tyrosine/Tryptophan (Tyr/Trp) were calculated. The duration of each mapping was 8 min. The sensitivity of UV microspectroscopy on tissue was on the order of micromolar. Autofluorescence spectra were deconvoluted using Labspec software (Jobin-Yvon, France). The use of synchrotron light as a source of UV permitted the excitation light to be tuned to correspond with the absorption of endogenous fluorochromes [5]. Correlated transmission images obtained on Synchrotron UV beamline are shown on figures **1** and **2**. Typical UV fluorescence spectra and their distribution on osteocyte and surrounding matrix ROIs are shown on figures **2** and **3**. UV chemical maps showing the tyrosine, tryptophan, and collagen distribution are shown on figure **3**.

### Statistics

Numerical variables were expressed as mean ± standard deviation (sd). The statistics were realised with the Staview 5.0 software. The normality of the distribution was tested with the Shapiro-Wilk test and the homogeneity of variance was tested with the Fisher F test. When the distribution of the data for each group respected the normality law and the variance of groups were homogeneous, t-test for paired measures was used. When the distribution of at least one of the groups did not follow the normality law, comparisons between groups were performed with the nonparametric Kruskall-Wallis test, followed with two-by-two comparisons performed with the U test of Mann and Whitney. Two groups of diseased rats were compared with those of the control group rats. If there were differences, a Bonferroni correction was applied for the comparison between the three groups and a *P* value of 0.016 or less was regarded as significant. The critical p-value for statistical significance was p = 0.05, for the ratios comparisons in each different group.

## Results

### Alcohol Consumption Induces a Decrease in BMD

At baseline, there was no whole body and femur BMD difference between the three groups. At the end of the protocol, whole body BMD was significantly lower in the A25 and A35 groups compared to C (table 1). Moreover, at the end of the protocol, femur BMD was significantly lower in the A25 and A35 groups compared to C (table 1).

**Table 1:** Bone mineral density, microarchitecture of the trabecular and cortical bone at the femur diaphysis after a bone animal experiment on chronic alcohol consumption.

	C (n = 11)	A25 (n = 8)	A35 (n = 8)	p
**WB BMD (g/cm2)**	0,207±0,004	0,194±0,005^c^	0,189±0,006^c^	p<0,0001
**Femur BMD(g/cm2)**	0,394±0,013	0,354±0,010^c^	0,335±0,020c,a	p<0,0001
**BV/TV (%)**	16,09±5,49	13,83±3,73	12,09±2,64	NS
**Tb.Th (mm)**	0,095±0,011	0,083±0,005^c^	0,085±0,009	0,03
**Ct.Po (%)**	1,03±0,28	1,15±0,32	1,42±0,35^c^	0,01
**Po.N (1/mm)**	0,24±0,05	0,29±0,07	0,35±0,08^c^	0,008
**PoS/CtV(mm^2^/mm^3^)**	1.63±0.17	1.91±0.34	2.24±0.54^c^	0,002

The alcohol-fed rats (A25, A35) drank alcohol for 17 weeks (25% and 35% v/v) while the control group (C) drank water. The critical p-value (p) was 0.05;

c significant according to controls,

a significant according to A25 group. Data were expressed as mean ± standard deviation (sd).

### Alcohol Consumption has Deleterious Effect on Bone Microarchitecture

At the end of the protocol, Tb.Th was significantly lower in the A25 group versus C **(** table 1). PoS/CtV, Ct.Po and Po.N were significantly higher in the A35 group compared to C at the end of the protocol (table 1).

### Synchrotron-UV Microspectroscopy

#### Three UV peaks are detected

An example of the ROIs investigated with UV beamline is indicated on figure **1**, whereas transmission images of cortical bone obtained with Synchrotron UV beamline are presented on figures **1**, **2**. The first observation of the UV fluorescence emission spectra is characterized by three peaks at the wavelengths 305, 333, 385 nm (figures **2** **,** **3**), attributed to tyrosine, tryptophan and collagen respectively [6], [8]. Figure **3** shows the mapping of these compounds by step of 2×2 µm in the osteocyte and its environment (voxel size 2×2×4 µm^3^).

#### A35 group presents the highest ratios

**Figure 4:**
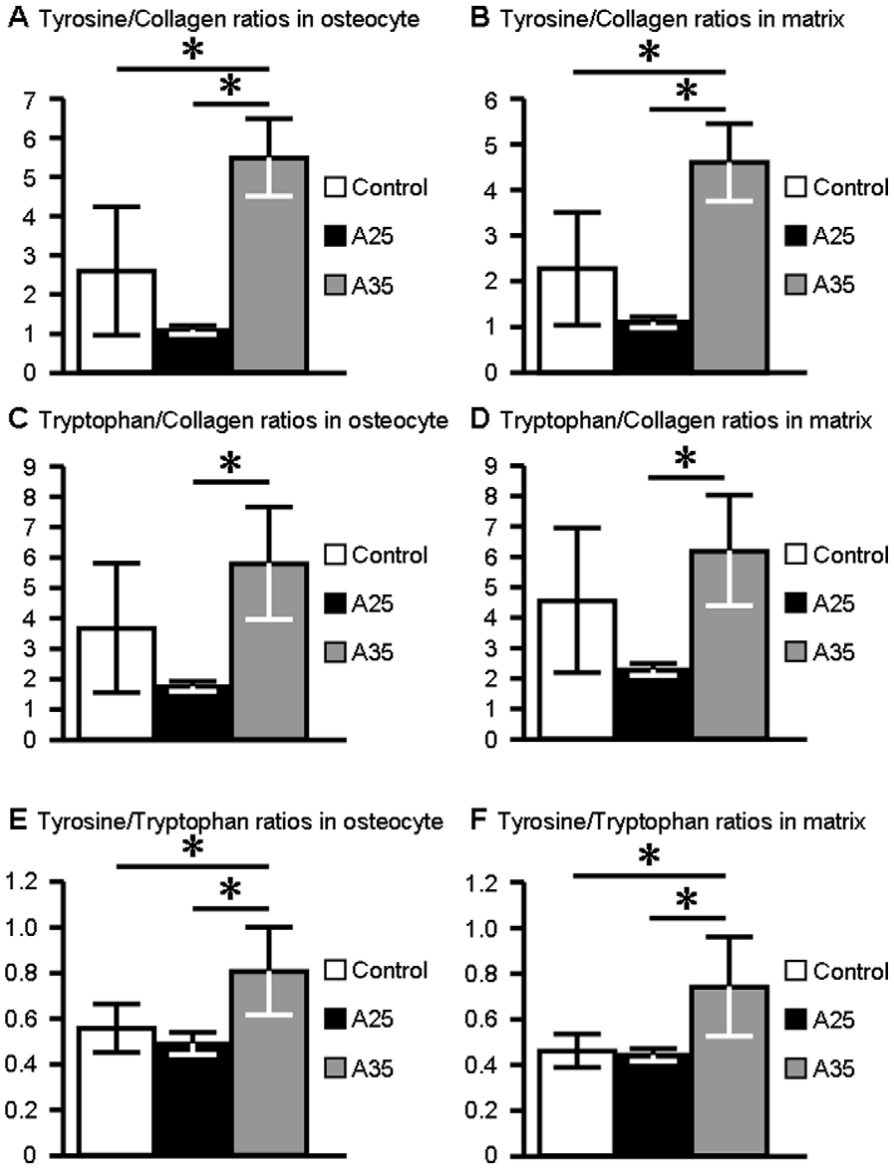
UV emission peak intensity ratios in osteocyte and surrounding matrix ROIs for the three different groups. **A**: Tyrosine/Collagen ratio in osteocyte ROI. **B**: Tyrosine/Collagen ratio in matrix ROI. **C**: Tryptophan/Collagen ratio in osteocyte ROI. **D**: Tryptophan/Collagen ratio in matrix ROI. **E**: Tyrosine/Collagen ratio in osteocyte ROI. **F**: Tyrosine/Collagen ratio in matrix ROI. shows a significant difference between the groups. Ratios are expressed as mean ± standard deviation (sd).

The tyrosine/collagen ratio in osteocyte ROI was significantly higher in the A35 group compared to C (4.86±0.88 and 2.30±1.44 for A35 and C; p = 0.008) (figure **4a**). The tyrosine/collagen ratio in the extracellular matrix ROI was also higher in the A35 group compared to C (4.61±0.85 and 2.28±1.23 for A35 and C; p = 0.001) (figure **4b**). The tyrosine/tryptophan ratio in osteocyte ROI was significantly higher in the A35 group compared to C (0.84±0.20 and 0.58±0.11 for A35 and C; p = 0.008) (figure **4e**). The tyrosine/tryptophan ratio in the extracellular matrix ROI was also significantly higher in the A35 group compared to C (0.82±0.24 and 0.51±0.08 for A35 and C; p = 0.010) (figure **4f**).

If we compare both alcohol consumptions, the tyrosine/collagen ratio in osteocyte ROI was significantly higher in the A35 group compared to A25 (respectively 4.86±0.88 and 0.97±0.10 for A35 and A25; p = 0.008) (figure **4a**). Moreover, the tyrosine/collagen ratio in the extracellular matrix ROI was also higher in the A35 group compared to A25 (respectively 4.61±0.85 and 1.11±0.12 for A35 and A25; p = 0.008) (figure **4b**).

The tryptophan/collagen ratio in osteocyte ROI was significantly higher in the A35 group compared to A25 (6.20±1.97 and 1.89±0.17 for A35 and A25; p = 0.008) (figure **4c**). The tryptophan/collagen ratio in the extracellular matrix ROI was also significantly higher in the A35 group compared to A25 (6.03±1.76 and 2.24±0.19 for A35 and A25; p = 0.008) (figure **4d**).

Furthermore, the tyrosine/tryptophan ratio in the osteocyte ROI was significantly higher in the A35 group compared to A25 (0.84±0.20 and 0.51±0.05; p = 0.001) (figure **4e**). The tyrosine/tryptophan ratio in the extracellular matrix ROI was also higher in the A35 group compared to A25 (0.82±0.24 and 0.49±0.03 for A35 and A25; p = 0.015) (figure **4f**).

There was no statistically significant difference for ratios in osteocyte and extracellular matrix ROI between controls and the A25 group (figure **4**).

#### Difference between Tryptophan/collagen and tyrosine/collagen ratios depends upon to the alcohol dose

**Figure 5:**
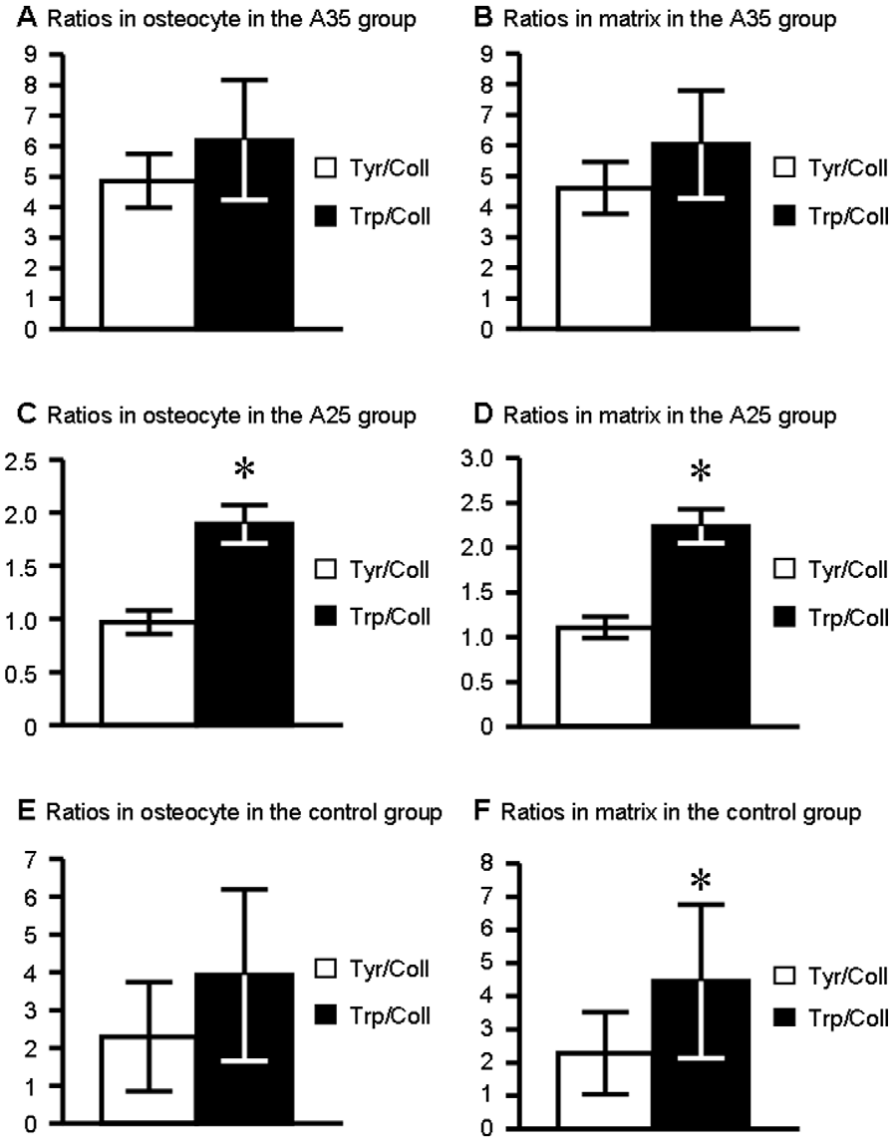
Tyrosine/Collagen and Tryptophan/Collagen ratios in osteocyte and surrounding matrix for the different groups. **A**: Comparison between Tyrosine/Collagen and Tryptophan/Collagen ratios in osteocyte ROI in A35. **B**: Comparison between Tyrosine/Collagen and Tryptophan/Collagen ratios in matrix ROI in A35. **C**: Comparison between Tyrosine/Collagen and Tryptophan/Collagen ratios in osteocyte ROI in A25. **D**: Comparison between Tyrosine/Collagen and Tryptophan/Collagen ratios in matrix ROI in A25. **E**: Comparison between Tyrosine/Collagen and Tryptophan/Collagen ratios on osteocyte ROI for controls. **F**: Comparison between Tyrosine/Collagen and Tryptophan/Collagen ratios on matrix ROI for controls. shows a significant difference between the ratios (p<0.05). Ratios are expressed as mean ± standard deviation (sd).

There was no statistically significant difference between tyrosine/collagen and tryptophan/collagen ratios in the osteocyte ROI (4.86±0.88 vs 6.20±1.97, p = 0.248) (figure **5a**) and extracellular matrix ROI (4.61±0.85 vs 6.03±1.76, p = 0.089) for the A35 alcohol samples (figure **5b**).

There was a statistically significant difference between tyrosine/collagen and tryptophan/collagen ratios both in the osteocyte ROI (0.97±0.11 vs 1.89±0.18 respectively, p<0.0001) and extracellular matrix ROI (1.11±0.12 vs 2.24±0.19, p<0.0001) for the A25 alcohol samples (figure **5c–d**).

#### Tryptophan/collagen ratio differs from the ROIs in the C group

For the control samples, the tryptophan/collagen ratio was not significantly different from the tyrosine/collagen ratio in the osteocytes (3.93±2.27 vs 2.30±1.44; p = 0.081) (figure **5e**). On the contrary, the tryptophan/collagen ratio was significantly higher than the tyrosine/collagen ratio in the extracellular matrix ROI (4.45±2.32 vs 2.28±1.23; p = 0.045) (figure **5f**).

There was a statistically significant difference for ratios between osteocyte and extracellular matrix ROI for the tryptophan/collagen ratio in the C group (osteocyte vs matrix: 3.93±2.27 vs 4.44±2.32, p = 0.020) (table **2**).

**Table 2:** Comparison of the UV spectroscopy Tyrosine/Collagen (Tyr/Coll) and Tryptophan/Collagen (Trp/Coll) ratios in osteocyte and matrix ROI for the A25 (n = 8), A35 (n = 8) and Control groups (n = 11).

	Tyr/Coll osteocyte ratio	Tyr/Coll matrix ratio	p
Control samples	2.30±1.44	2.28±1.23	NS
A25 samples	0.96±0.10	1.11±0.12	p = 0.002
A35 samples	4.86±0.88	4.61±0.85	NS
	**Trp/Coll osteocyte ratio**	**Trp/Coll matrix ratio**	**p**
Control samples	3.93±2.27	4.44±2.32	p = 0.020
A25 samples	1.89±0.18	2.24±0.20	p<0.0001
A35 samples	6.20±1.97	6.03±1.76	NS

NS: non significant difference between ratios. Data were expressed as mean ± standard deviation (sd).

#### Comparison of osteocyte and matrix ratios in alcohol groups

**Figure 6:**
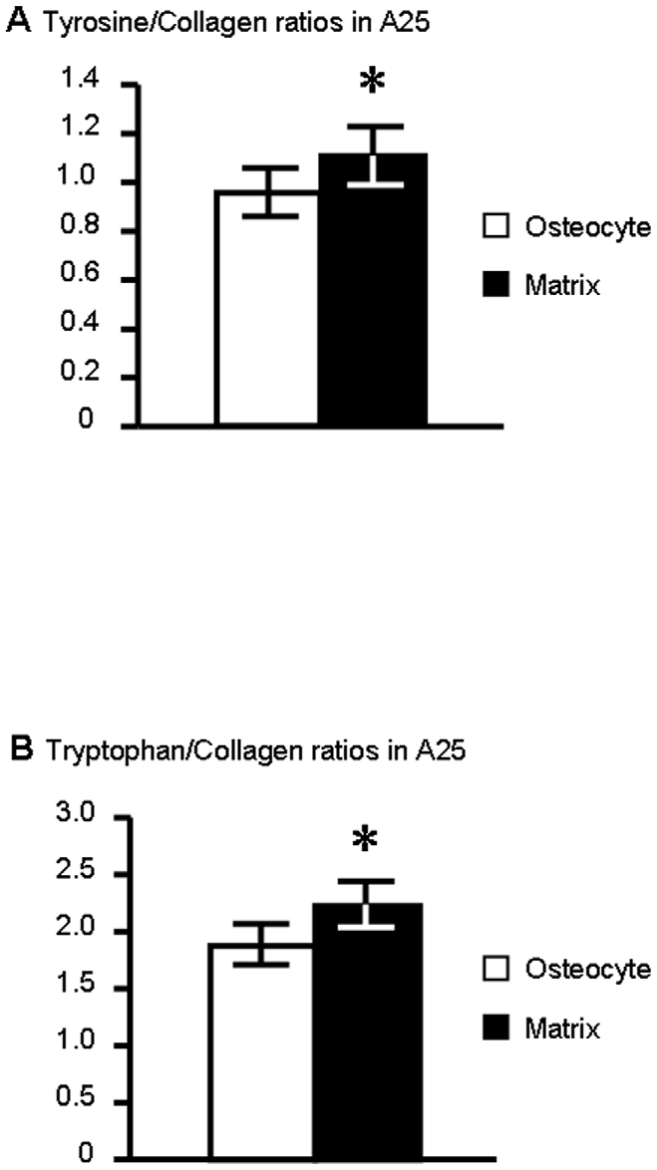
Tyrosine/Collagen and Tryptophan/Collagen ratios on osteocyte and surrounding matrix for the A25 group. **A**: Comparison between osteocyte and matrix for the Tyrosine/Collagen ratio. **B**: Comparison between osteocyte and matrix for the Tryptophan/Collagen ratio. shows a significant difference between the ratios (p<0.05). Ratios are expressed as mean ± standard deviation (sd).

If we compare the osteocyte and its surrounding matrix, we obtain a statistically significant difference in the A25 group for the tyrosine/collagen ratio (osteocyte vs matrix : 0.96±0.10 vs 1.11±0.12, p = 0.0025) (table **2** **,** figure **6**) and for the tryptophan/collagen ratio (osteocyte vs matrix : 1.89±0.18 vs 2.24±0.20, p<0.0001) (table **2** **,** figure **6**). There was no statistically significant difference for ratios between osteocyte and extracellular matrix ROIs for the A35 group.

## Discussion

This study has shown that synchrotron UV microspectroscopy may give quantitative information on the cortical bone content of some autofluorescent molecules in osteocytes and bone extracellular matrix. Here, the biological model was focused on alcohol-induced osteoporosis [25]. Our first results in term of BMD and cortical microarchitecture measurements have confirmed deleterious effects of chronic alcohol consumption on bone.

Synchrotron UV spectroscopy is a promising tool which permit to identify biochemical targets which naturally absorb in the UV and deep UV. This autofluorescence could follow up metabolic processes involved in the field of osteoarticular diseases. This powerfull tool can contribute to follow these targets both in the osteocyte and the surrounding matrix *in situ* and to obtain a quantitative distribution of these molecules. This methodology allows to distinguish between osteocyte and surrounding matrix, since osteocytes are considered as the orchestrator of the bone remodelling and mineralization around the lacunae [10], [11].

This tool is very innovative because these natural autofluorescent probes are involved in the bone cell physiology and variations of their distribution could be measured under different osteoarticular physiopathological contexts (osteoporosis, osteoarthritis, Apert Syndrome …).

Our first UV Synchrotron microspectroscopy data showed that bone samples express three peaks at the wavelengths 305, 333, 385 nm, which correspond to tyrosine, tryptophan and collagen, respectively. Second, we have also observed that tyrosine/collagen and tryptophan/collagen ratios were significantly higher in heavy alcohol consumption group (figure **4**).

We did not observe any significant difference for tyrosine/collagen, tryptophan/collagen and tyrosine/tryptophan ratios between C and A25 groups. As a consequence, we did not observe a dose effect response on these parameters.

Third, we have observed that significant difference between tryptophan/collagen and tyrosine/collagen ratios is dependent upon the alcohol dose.

Fourth, we have noticed that these ratios are different dependent upon the site of analysis: whether in the osteocyte or in the surrounding matrix (figures **4** **,** **5** **,** **6** **,** table **2**). Only in the moderate alcohol consumption group (25% v/v), tryptophan/collagen and tyrosine/collagen ratios were higher in matrix than in osteocyte (table **2**). Fifth, only strong chronic alcohol consumption induced an increase of the ratio Tyrosine/Tryptophan both in cell and surrounding matrix. To our knowledge, this is the first characterization *in situ* of the tyrosine/collagen and tryptophan/collagen contents in bone sections, both in cell and matrix environments.

The involvement of tyrosine and tryptophan in an alcohol-induced osteoporosis model is not well known, whereas the role of tryptophan in different metabolic pathways has been highlighted [16], [17], [32]. Measurement of the tryptophan and the tryptophan/collagen ratio could give informations related to the serotonin metabolism. This metabolism is considered as an element involved in the bone remodeling regulation particularly in bone formation [17]. Indeed, Bliziotes *et al.*, 2006, have shown that the rate-limiting enzyme for serotonin synthesis, the tryptophan hydroxylase is expressed in MLO-Y4 cell lines that are considered to be osteocyte lines [33]. Furthermore, they have demonstrated that osteocytes, as well as osteoblasts, are capable of serotonin (5-HT) synthesis, and functional receptor expression [33]. The involvement of 5-HT in stress-induced alcohol-related behaviours is particularly interesting in view of the hypothesis that reduced serotonergic function may contribute to the development of alcoholism [34]. More recent findings from genomic studies have also shown a causal link between 5-HT transporter promoter polymorphism and susceptibility to alcoholism [35]. A previous work [36] used a dietary tryptophan enhancement method to explore behavioral effects in alcoholic individuals who already suffer from a 5-HT dysfunction.

We note that significant differences between moderate and high alcohol consumption samples in the tryptophan/collagen ratio (figure **4c–d**) could suggest that the tryptophan hydroxylase metabolic pathway is involved in the osteocyte response to the high alcohol consumption. Tryptophan is also involved in vitamin D receptor action. Nguyen *et al.*, 2002, have shown that a tryptophan missense mutation in the ligand-binding domain of the vitamin D receptor (and/or a substitution of the tryptophan by arginine) in fibroblasts as well as COS-7 cells, causes severe resistance to 1,25-Dihydroxyvitamin D [18].

Among bone cell metabolic pathways in which tryptophan is involved, Lemonnier *et al.*, 2000 [19], have demonstrated that in Apert Syndrome, a form of acrocephalosyndactyly characterized by premature ossification and fusion of cranial sutures, the origin of the pathology is the Ser252Trp mutation of the fibroblast growth factor receptor FGFR2. In the field of bone biomaterials, ordered mesoporous silica-based bioceramics have been evaluated for their capacity to uptake and deliver L-tryptophan [37]. This amino-acid corresponds to the end position of the 107–111 domain (called osteostatin) of the native C-terminal PTHrP (107–139) fragment. This osteostatin domain can inhibit osteoclast bone resorption [38], [39] and stimulate bone formation [20], [21].

Lozano *et al*., 2010, have demonstrated that osteostatin-loaded bioceramics stimulate osteoblastic growth and differentiation [37]. In this field, our results have shown that the tryptophan/collagen ratio is statistically higher in A35 vs A25 both in osteocyte and matrix. Our previous data [13] have shown that resorption markers were higher in A35 vs A25 (NTx: 31.53±5.75 vs 13.41±3.26 nM). Here, the upper expression of tryptophan could represent a feedback loop consequence to counteract bone resorption; but this remains a hypothesis.

Some previous works highlight the role of tyrosine in different bone cell metabolic pathways [40]–[47] but the exact role of the residue tyrosine in osteoporosis models is not well documented. It is involved in Protein Tyrosine Kinases/Protein Tyrosine Phosphatases (PTKs/PTPs) transduction signaling pathways which play a role in the control of the osteoblast/osteocyte metabolism [10]. These PTKs/PTPs are involved in the regulation of bone formation. Indeed, Billiard *et al*., 2005, have demonstrated that the Orphan Receptor Tyrosine Kinase Ror2 plays a crucial role in skeleton developmental morphogenesis [40]. They have shown that human Ror2 is a regulator of canonical Wnt signalling in osteoblastic lineage which has physiological consequences in bone, *via* modulation of osteoblast survival and differentiation.

Schinke *et al.*, 2008, have highlighted that the PTP Rptpζ is not only expressed in differentiated osteoblasts but also affects bone formation in mice [41]. The PTP superfamily contains more than 100 members, and few of them have been previously suggested to play a role in skeletal development and metabolism [42]. Given the general importance of PTPs in the regulation of osteoblast proliferation and differentiation, the action of specific members of the PTP superfamily might be involved in the control of bone formation and resorption. As previously noticed by Schinke *et al*., 2008, the first PTP that has been demonstrated to play a physiological role in bone remodelling is Shp1 [41]. Thus, Shp1 was identified as a negative regulator of osteoclastogenesis [43].

An other PTP known as osteotesticular protein tyrosine phosphatase (OST-PTP), has been involved in bone formation [44]. *Ptprv*, the gene encoding OST-PTP has been described specifically expressed in osteoblasts and gonads. Schinke *et al*., 2008, have shown that it appears that the activity of OST-PTP is potentially required at an earlier stage of osteoblast differentiation, unlike the activity of Rptpζ, whose expression is only detectable in fully differentiated and mineralized osteoblast cultures [41].

The literature illustrates the involvement of PTKs and tyrosine phosphorylation of proteins such p^125^FAK or paxillin in osteoblast/osteocyte differentiation and adhesion to extracellular matrix [45]–[47]. Tyrosine-derived polycarbonate membranes were also proposed as a biomaterial to treat mandibular bone defects [48].

Our data have shown that the tyrosine/collagen ratio is strongly increased in the high alcohol consumption group, which could suggest that the balance between PTKs and PTPs activity in osteocytes can be modulated by the level of alcohol consumption; and as a consequence, the bone remodelling could be modified. Alcohol-induced osteoporosis is known to be mainly linked to a decrease in bone formation [12] with an increase in bone resorption in some cases [13].

In conclusion, synchrotron UV microspectroscopy constitutes a new method of cell characterization *in situ* which allows evaluating osteocyte metabolism and gives a better understanding of the key role of this cell in the bone remodeling regulation. This local measurement of tyrosine and tryptophan level may give a better understanding of cellular metabolism according to different physio-pathological conditions.
